# South African Propolis: Anti‐*Helicobacter pylori* Activity, Chemistry, and Toxicity

**DOI:** 10.1002/cbdv.202403200

**Published:** 2025-04-01

**Authors:** Sarhana Dinat, Ané Orchard, Efficient Ncube, Weiyang Chen, Alvaro Viljoen, Sandy van Vuuren

**Affiliations:** ^1^ Department of Pharmacy and Pharmacology, Faculty of Health Sciences University of the Witwatersrand Johannesburg South Africa; ^2^ Department of Pharmaceutical Sciences, Faculty of Sciences Tshwane University of Technology Pretoria South Africa; ^3^ SAMRC Herbal Drugs Research Unit, Department of Pharmaceutical Sciences Tshwane University of Technology Pretoria South Africa

**Keywords:** biological activity, *Helicobacter pylori*, liquid chromatography, propolis, toxicity

## Abstract

Propolis, a resin‐like substance produced by bees, has previously shown antimicrobial activity against the ulcer‐causing gut pathogen *Helicobacter pylori*. South African propolis, however, was yet to be investigated. This study aimed to investigate a comprehensive range of South African propolis for its antimicrobial activity against *H. pylori* and to investigate toxicity. A total of 51 samples were collected from around South Africa and comparatively analysed with three Brazilian samples. The antimicrobial broth microdilution assay was used to determine the minimum inhibitory concentration (MIC) of ethanolic propolis extracts against three clinical *H. pylori* strains. A total of 27 South African propolis extracts presented antimicrobial activity better than that of the Brazilian samples (MIC ≤ 0.51 mg/mL). Samples with the best anti‐*H. pylori* activity were selected for chemical analysis using ultra‐performance liquid chromatography–mass spectrometry. The compounds pinocembrin, 3‐*O*‐acetylpinobanksin, and pinobanksin were found to be the most abundant. All propolis extracts investigated in this study were considered non‐toxic (mortality < 50%) when investigated using the brine shrimp lethality assay. This study demonstrates the in vitro potential of utilizing propolis for treating *H. pylori* infections and highlights the possible compounds responsible for the activity observed.

## Graphical abstract

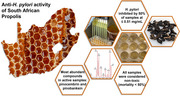

## 1 Introduction

*Helicobacter pylori* colonizes the human gastric mucosa and has been found to infect over 50% of the global population, with prevalence in South Africa ranging between 50% and 84% [1]. It is implicated in a variety of gastric ailments, most notably causing gastric ulcers [2]. Treatment options for *H. pylori* infections currently include proton pump inhibitors, bismuth and multiple antibiotics, such as amoxicillin and clarithromycin [3]. As *H. pylori* rapidly gains resistance to antibiotics, treatment options are increasingly losing their efficacy, with increasing resistance rates to clarithromycin reported in South Africa [4]. Thus, natural products serve as an attractive alternative treatment for *H. pylori* infections and related ailments.

Propolis, a resin‐like substance, is produced by the *Apis mellifera* bee and used to repair and reinforce the hive. It also acts as an antiseptic, preventing infections within the hive [5]. The medicinal use of propolis has been recorded for thousands of years and has been used historically in embalming and wound healing [6, 7]. Propolis has since been shown to have numerous medicinal properties, including anti‐ulcer, anti‐inflammatory, anti‐tumour and antimicrobial activity [7, 8, 9, 10]. Propolis has been noted as a valuable anti‐*H. pylori* agent, with studies investigating anti‐ulcer activity, enzyme inhibition and anti‐oxidant activity, as well as antimicrobial activity [11, 12].

Several studies have investigated the anti‐ulcer and antimicrobial activity against *H. pylori* of propolis that were collected from different regions around the world, including Spain, Korea and Chile [11, 12]. Brazilian propolis, commercially highly regarded, is noted for a wide range of medicinal and health benefits and was also shown to have anti‐*H. pylori* activity [12, 13]. African propolis, however, has been poorly explored for its anti‐*H. pylori* activity. Only two studies were carried out in the past 10 years, both investigating Nigerian propolis, with the results indicating good antimicrobial activity [14, 15, 16]. Southern African propolis, however, is yet to be explored in this context. South African propolis was studied for its antimicrobial activity against a range of pathogens, with various propolis samples from South Africa and Brazil shown to have substantial antimicrobial activity against *Staphylococcus aureus* [7]. A South African propolis sample, along with three associated propolis compounds, pinocembrin, galangin and chrysin, were investigated against a variety of bacterial and fungal pathogens, where combinations of the compounds showed the best antimicrobial activity [17]. The anti‐*H. pylori* potential of South African propolis, however, was yet to be investigated.

The pharmacological activity of propolis is influenced by the chemical composition, which varies depending on the geographic location, season of collection, bee species and plant sources [11]. Over 300 compounds have been identified from propolis samples, including polyphenols, terpenoids, steroids, and amino acids. Flavonoids, such as pinocembrin, acacetin, and chrysin, were found to be the most abundant [10]. Antimicrobially active South African propolis was reported to be primarily comprised of pinocembrin, galangin, and chrysin. The chemical profiles noted for South African propolis were similar to that of temperate region propolis, and were distinct from Brazilian propolis, of the tropical regions [7].

While natural products are often assumed to be harmless and safe for consumption, several studies have shown that natural products can be hazardous and possibly lethal [18]. The toxicity of Southern African propolis is yet to be explored, with only one study investigating the toxicity of a single whole propolis sample and the compounds pinocembrin, galangin and chrysin [17]. Thus, this study aimed to investigate a range of South African propolis samples for their antimicrobial activity against *H. pylori*, investigate the toxicity, and provide a chemical profile of samples demonstrating good antimicrobial activity using ultra‐performance liquid chromatography–mass spectrometry (UPLC‐MS).

## 2 Results and Discussion

### 2.1 Antimicrobial Activity

The minimum inhibitory concentration (MIC) values (*n* = 3) of 54 propolis ethanolic extracts tested against three clinical *H. pylori* strains are reported in Table 1. Good antimicrobial activity was recorded for the MIC values that were less than or equal to the mean MIC across all three Brazilian samples, 0.51 mg/mL. Good anti‐*H. pylori* activity was observed against at least one *H. pylori* strain for 27 South African samples, with notable mean MIC values across all three strains recorded for 19 propolis extracts. Good anti‐*H. pylori* activity was recorded for 15 samples from the Gauteng Province, six samples from the Western Cape, three samples from the Northern Cape and one sample each from Free State, Kwa‐Zulu Natal and Limpopo. The best activity was seen in samples 13 (Gauteng), 44 (Western Cape) and 37 (Northern Cape), with mean MIC values of 0.20, 0.20 and 0.23 mg/mL, respectively.

**TABLE 1:** The MIC values in mg/mL of South African propolis extracts against three clinical *Helicobacter pylori* strains represented as mean ± standard deviation.

Propolis sample	Location	Province of origin	Clinical Strain 1	Clinical Strain 2	Clinical Strain 3	Mean
1	Port Elizabeth—Baviaanskloof	Eastern Cape	> 6.25 ± 0.00	> 6.25 ± 0.00	> 6.25 ± 0.00	> 6.25 ± 0.00
2	Bloemfontein	Free State	**0.39** ^a^ ± 0.00	0.59 ± 0.00	0.78 ± 0.00	0.59 ± 0.28
3	Benoni—Bapsfontein	Gauteng	0.78 ± 0.00	1.56 ± 0.00	**0.39** ± 0.00	0.91 ± 0.43
4	Bronkhorstspruit—Wilgerivier	Gauteng	0.78 ± 0.00	0.78 ± 0.00	**0.29** ± 0.14	0.62 ± 0.19
5	Edenvale	Gauteng	**0.20** ± 0.14	**0.39** ± 0.00	**0.20** ± 0.00	**0.26** ± 0.23
6	Edenvale	Gauteng	**0.29** ± 0.00	0.78 ± 0.00	**0.20** ± 0.00	**0.42** ± 0.19
7	Johannesburg	Gauteng	**0.39** ± 0.00	0.78 ± 0.00	**0.39** ± 0.00	0.52 ± 0.43
8	Johannesburg	Gauteng	0.78 ± 0.00	0.78 ± 0.00	0.78 ± 0.00	0.78 ± 0.28
9	Johannesburg	Gauteng	0.78 ± 0.00	0.78 ± 0.00	0.78 ± 0.00	0.78 ± 0.00
10	Lakeside/Westlake	Gauteng	0.78 ± 0.00	1.56 ± 0.00	**0.39** ± 0.00	0.91 ± 0.47
11	Midrand—Beaulieu	Gauteng	**0.10** ± 0.00	0.98 ± 0.00	**0.10** ± 0.00	**0.39** ± 0.37
12	Midrand—President Park	Gauteng	**0.39** ± 0.00	**0.39** ± 0.00	**0.39** ± 0.00	**0.39** ± 0.18
13	Pretoria	Gauteng	**0.20** ± 0.00	**0.20** ± 0.00	**0.20** ± 0.00	**0.20** ± 0.25
14	Pretoria	Gauteng	0.78 ± 0.00	3.13 ± 0.00	0.78 ± 0.00	1.56 ± 0.97
15	Pretoria	Gauteng	**0.39** ± 0.00	**0.39** ± 0.00	**0.39** ± 0.00	**0.39** ± 0.10
16	Pretoria East	Gauteng	> 6.25 ± 0.00	> 6.25 ± 0.00	> 6.25 ± 0.00	> 6.25 ± 0.00
17	Pretoria—Groenkloof	Gauteng	> 6.25 ± 0.00	> 6.25 ± 0.00	> 6.25 ± 0.00	> 6.25 ± 0.00
18	Pretoria—Lydiana Gardens	Gauteng	0.78 ± 0.00	**0.39** ± 0.00	0.59 ± 0.00	0.59 ± 0.26
19	Pretoria—Lydiana Gardens	Gauteng	**0.20** ± 0.00	0.78 ± 0.00	**0.20** ± 0.00	**0.39** ± 0.29
20	Pretoria—Northern	Gauteng	6.25 ± 0.00	> 6.25 ± 0.00	> 6.25 ± 0.00	> 6.25 ± 0.00
21	Sandton	Gauteng	1.56 ± 0.00	3.13 ± 0.00	2.35 ± 0.00	2.35 ± 2.13
22	Sandton	Gauteng	0.78 ± 0.00	1.56 ± 0.00	1.56 ± 0.00	1.30 ± 0.51
23	Sandton	Gauteng	3.13 ± 0.00	3.13 ± 0.00	2.35 ± 0.00	2.87 ± 2.66
24	Sandton—IDC Grayston Drive	Gauteng	1.56 ± 0.00	3.13 ± 0.00	1.56 ± 0.00	2.08 ± 1.04
25	Sandton—Paulshof	Gauteng	**0.39** ± 0.00	**0.39** ± 0.00	**0.39** ± 0.00	**0.39** ± 0.55
26	Sandton—Riverclub	Gauteng	6.25 ± 0.00	6.25 ± 0.00	6.25 ± 0.00	6.25 ± 2.74
27	Sandton—Woodlands Eco Park	Gauteng	1.56 ± 0.00	3.13 ± 0.00	3.13 ± 0.00	2.61 ± 0.82
28	Sedibeng area—Devon	Gauteng	0.78 ± 0.00	0.78 ± 0.00	**0.39** ± 0.00	0.65 ± 0.28
29	Springs	Gauteng	**0.20** ± 0.00	**0.39** ± 0.00	**0.20** ± 0.00	**0.26** ± 0.25
30	Vereeniging—Walkerville	Gauteng	**0.20** ± 0.00	0.78 ± 0.00	**0.20** ± 0.00	**0.39** ± 0.30
31	SANS	Kwa‐Zulu Natal	**0.39** ± 0.00	**0.39** ± 0.00	**0.39** ± 0.00	**0.39** ± 0.31
32	Amandelbult	Limpopo	**0.39** ± 0.00	0.78 ± 0.00	**0.39** ± 1.11	0.52 ± 1.12
33	Naboomspruit and Nylstroom	Limpopo	**0.39** ± 0.00	**0.20** ± 0.00	**0.20** ± 0.00	**0.26** ± 0.08
34	Mooinooi	North West	> 6.25 ± 0.00	> 6.25 ± 0.00	> 6.25 ± 0.00	> 6.25 ± 0.00
35	Douglas	Northern Cape	**0.39** ± 0.00	0.78 ± 0.00	0.59 ± 0.28	0.59 ± 0.18
36	SANS	Northern Cape	**0.39** ± 0.00	0.78 ± 0.14	0.59 ± 0.00	0.59 ± 0.23
37	SANS	Northern Cape	**0.20** ± 0.00	**0.29** ± 0.00	**0.20** ± 0.28	**0.23** ± 1.45
38	SANS	Northern Cape	> 6.25 ± 0.00	> 6.25 ± 0.00	> 6.25 ± 0.00	> 6.25 ± 0.00
39	Orange River	Unknown	1.56 ± 0.00	3.13 ± 0.00	2.35 ± 1.11	2.35 ± 0.86
40	Beaufort West	Western Cape	0.78 ± 0.00	0.78 ± 0.00	0.78 ± 0.00	0.78 ± 0.00
41	Botrivier	Western Cape	0.78 ± 0.00	0.78 ± 0.00	0.78 ± 0.00	0.78 ± 0.31
42	Cape Town—Southern Suburbs	Western Cape	**0.39** ± 0.00	**0.20** ± 0.00	**0.20** ± 0.00	**0.26** ± 1.07
43	Cape Town—Southern Suburbs	Western Cape	**0.10** ± 0.00	0.78 ± 0.00	**0.10** ± 0.00	**0.33** ± 0.25
44	Cape Town—Southern Suburbs	Western Cape	**0.20** ± 0.00	**0.20** ± 0.00	**0.20** ± 0.00	**0.20** ± 0.35
45	Graafwater	Western Cape	0.78 ± 0.00	0.78 ± 0.00	0.78 ± 0.00	0.78 ± 0.29
46	Outeniqua Mountains—Oudtshoorn	Western Cape	**0.20** ± 0.00	**0.39** ± 0.00	**0.20** ± 0.00	**0.26** ± 0.11
47	SANS	Western Cape	**0.39** ± 0.00	0.78 ± 0.28	**0.20** ± 0.00	**0.46** ± 0.25
48	SANS	Western Cape	**0.20** ± 0.00	**0.39** ± 0.00	**0.20** ± 0.00	**0.26** ± 0.25
49	Somerset West	Western Cape	0.78 ± 0.00	0.78 ± 0.00	0.78 ± 0.00	0.78 ± 0.28
50	Stanford	Western Cape	1.56 ± 0.00	1.56 ± 0.00	1.17 ± 0.55	1.43 ± 0.85
51	Touwsrivier	Western Cape	0.78 ± 0.00	1.56 ± 0.00	0.78 ± 0.00	1.04 ± 0.40
B1	Brazil	—	**0.39** ± 0.00	0.78 ± 0.00	**0.39** ± 1.11	0.52 ± 0.77
B2	Brazil	—	0.10 ± 0.00	0.10 ± 0.00	**0.10** ± 0.00	**0.10** ± 0.48
B3	Brazil	—	1.17 ± 0.00	0.78 ± 0.00	0.78 ± 0.55	0.91 ± 0.32
	Controls					
	Amoxicillin (µg/mL)		0.31 ± 0.00	0.31 ± 0.00	0.16 ± 0.00	0.26 ± 0.08
	Acetone		> 6.25 ± 0.00	> 6.25 ± 0.00	> 6.25 ± 0.00	> 6.25 ± 0.00
	Culture control		growth	growth	growth	growth

Abbreviation: SANS, specific area not specified.

^a^ Good antimicrobial activity is denoted in bold.

While the antimicrobial activity of South African propolis against *H. pylori* has not been previously investigated, South African propolis extracts have previously shown promising antimicrobial activity against other Gram‐negative bacteria, where MIC values of 0.13, 0.16 and 0.21 mg/mL were observed against *Pseudomonas aeruginosa*, *Escherichia coli* and *Klebsiella pneumoniae*, respectively [17].

Propolis from Nigeria was found to possess anti‐*H. pylori* activity when assessed using the MIC and disc‐well assays [14, 15]. Similar inhibition was found for both clinical and reference strains, where MIC values recorded for propolis were eightfold higher than the positive control, amoxicillin [15]. In a separate study, Nigerian propolis was investigated against *H. pylori* isolated from gastric biopsy patients, and zones of inhibition of 30.00 and 23.00 mm were observed for propolis at concentrations of 400.00 and 200.00 µL/mL, respectively [14].

Propolis from northern Spain was investigated against *H. pylori* with ethanolic and propylene glycol extracts which were found to have MIC values ranging between 6.00 and 14.00 mg/mL [19]. These findings support the results of this study, indicating that propolis does possess antimicrobial activity against *H. pylori*. The results further confirm that variation in biological activity does exist from region to region, as the chemical distinction of propolis is region‐specific.

### 2.2 Chemical Analysis

**FIGURE 1:**
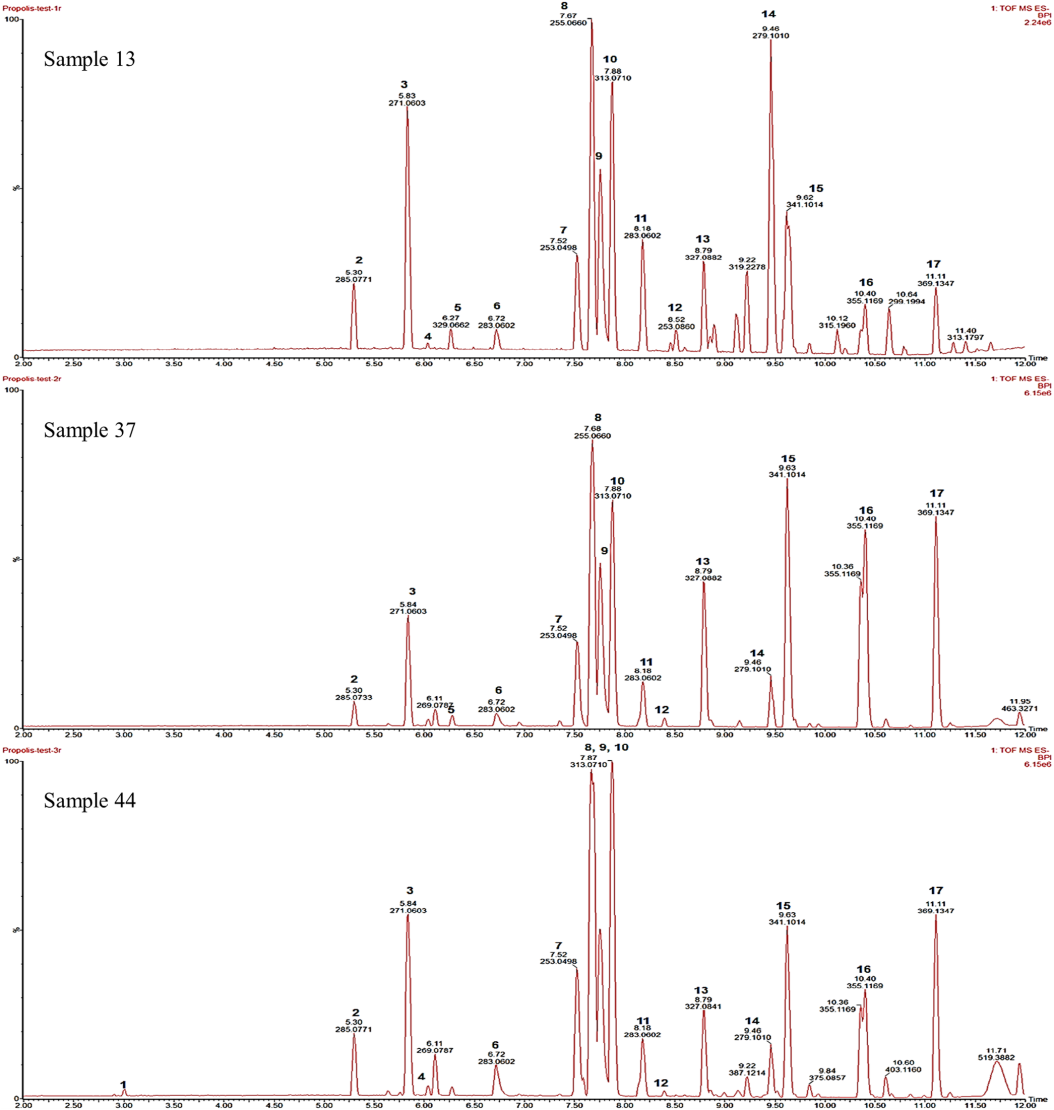
UPLC‐MS chromatograms of samples with the best anti‐*Helicobacter pylori* activity.

Samples 13, 37 and 44 displayed the best antimicrobial activity, with mean MIC values of 0.20, 0.23 and 0.20 mg/mL, respectively, and were thus selected for chemical analysis using UPLC‐MS. Liquid chromatography coupled with MS, particularly in the negative mode, allows for fast identification and accurate quantification of flavonoids that are ubiquitous in propolis [10, 20, 21]. Compounds were identified by matching retention times (Rt), mass–charge values (*m*/*z*), and fragmentation patterns to data available in literature and databases (Table 2). The chromatographic profiles observed (Figure 1) correlate with that previously reported for South African propolis and showed a similar profile to that of common temperate propolis [22].

**TABLE 2:** Compounds identified in ethanolic extracts of propolis samples 13, 37 and 44 using UPLC‐MS.

Peak #	Rt (min)	Elemental formula	[M−H]^−^ calculated	[M−H]^−^ theoretical	Mass error (mDa)	Fragments	Compound ID
1	3.05	C_9_H_8_O_3_	163.0395	163.0367	3.4	—	*p*‐Coumaric acid
2	5.30	C_16_H_14_O_5_	285.0763	285.0713	4.5	267, 267, 252, 239, 139	Pinobanksin‐methyl ether
3	5.84	C_15_H_12_O_5_	271.0606	271.0558	−4.9	253	Pinobanksin
4	6.04	C_16_H_11_O_6_	299.0556	299.0499	−5.7	284	Kaempferol‐3‐methyl ether
5	6.27	C_17_H_14_O_7_	329.0601	329.0761	−6.0	314, 299, 271	Dimethoxyquercetin
6	6.72	C_16_H_12_O_5_	283.0606	283.0554	−5.2	268, 239, 211	5‐Methoxygalangin
7	7.52	C_15_H_10_O_4_	253.0407	253.0454	4.7	145	Chrysin
8	7.67	C_15_H_12_O_4_	255.0657	255.0593	−6.4	213, 151	Pinocembrin
9	7.76	C_15_H_10_O_5_	269.0450	269.0409	−4.1	227	Galangin
10	7.88	C_17_H_14_O_6_	313.0712	313.0674	−3.8	253	3‐*O*‐Acetylpinobanksin
11	8.18	C_16_H_12_O_5_	283.0606	283.0554	−5.2	268, 239, 211	5‐Methoxygalangin
12	8.52	C_15_H_10_O_4_	253.0407	253.0454	4.7	145	Chrysin
13	8.79	C_18_H_16_O_6_	327.0869	327.0817	−5.2	271, 253	Pinobanksin‐3‐*O*‐propionate
14	9.46	C_18_H_16_O_3_	279.1021	279.0953	−6.8		*p*‐Cinnamyl coumarate
15	9.62	C_19_H_18_O_6_	341.1025	341.0973	−5.2	271, 253	Pinobanksin‐3‐*O*‐butyrate
16	10.40	C_16_H_20_O_9_	355.1029	355.1127	9.8	253	Pinobanksin‐3‐*O*‐pentanoate
17	11.11	C_21_H_22_O_6_	369.1338	369.1277	−6.1	271, 253	Pinobanksin‐3‐*O*‐hexanoate

A total of 17 dominant compounds were identified from the samples, with pinocembrin, 3‐*O*‐acetylpinobanksin and pinobanksin most abundant. The compounds chrysin, *p*‐coumaric acid, pinobanksin‐methyl ether, pinobanksin‐3‐*O*‐propionate, pinobanksin‐3‐*O*‐butyrate, pinobanksin‐3‐*O*‐pentanoate, and pinobanksin‐3‐*O*‐hexanoate had previously been identified from South African propolis samples [22, 23]. While the compounds kaempferol‐3‐methyl ether, dimethoxyquercetin, 5‐methoxygalangin, 3‐*O*‐acetylpinobanksin, and *p*‐cinnamyl coumarate were not previously identified in South African propolis, they were previously identified in South American, North American, Chinese and Mexican propolis, respectively [24, 25, 26, 27, 28].

It is plausible that the antimicrobial activity seen against *H. pylori* can be attributed mostly to pinocembrin, galangin and pinobanksin. These compounds have previously been noted for anti‐*H. pylori* activity [29]. The compounds chrysin, pinocembrin, galangin and caffeic acid phenyl ester were reported to show varying anti‐*H. pylori* activity, with caffeic acid phenyl ester being the most active, and synergy observed for the combination of chrysin with galangin [30]. Pinocembrin and galangin were previously reported to inhibit urease, an enzyme utilised by *H. pylori* during growth and colonization [2, 31]. Inhibition of urease by these compounds could be attributed to the anti‐*H. pylori* activity displayed by propolis.

### 2.3 Toxicity

The toxicity of the investigated propolis extracts is summarized in Table 3. All propolis extracts investigated in this study were considered non‐toxic, with a percentage mortality of less than 50%. Brazilian propolis extracts were also non‐toxic, with an average mortality of 0.79% at 24 h and 3.73% at 48 h, for all three samples.

**TABLE 3:** The toxicity of propolis extracts shown as mean ± standard deviation percentage mortality at 24 and 48 h of exposure.

Propolis sample	Mean percentage mortality	
	24 h	48 h
1	0.00 ± 0.00	0.00 ± 0.00
2	0.00 ± 0.00	26.94 ± 1.36
3	0.72 ± 1.26	2.35 ± 2.44
4	1.08 ± 7.86	24.92 ± 2.74
5	7.20 ± 9.99	26.32 ± 1.55
6	6.58 ± 2.40	29.63 ± 4.07
7	0.52 ± 0.90	40.08 ± 0.95
8	0.79 ± 1.37	0.79 ± 1.37
9	1.12 ± 0.00	34.80 ± 2.68
10	1.55 ± 1.35	12.62 ± 7.87
11	0.00 ± 0.00	38.03 ± 4.10
12	14.30 ± 5.79	20.89 ± 2.71
13	0.00 ± 0.00	0.68 ± 1.18
14	0.00 ± 0.00	0.00 ± 0.00
15	9.98 ± 5.05	30.29 ± 1.17
16	0.00 ± 0.00	10.09 ± 1.09
17	0.76 ± 0.00	3.22 ± 2.06
18	0.90 ± 1.56	0.90 ± 1.56
19	0.00 ± 0.00	2.04 ± 1.79
20	0.00 ± 0.00	3.26 ± 3.61
21	0.81 ± 1.41	0.81 ± 1.41
22	0.00 ± 0.00	0.00 ± 0.00
23	0.00 ± 0.00	0.65 ± 1.13
24	0.00 ± 0.00	0.00 ± 0.00
25	0.00 ± 0.00	2.94 ± 3.22
26	0.00 ± 0.00	2.73 ± 2.37
27	0.00 ± 0.00	4.74 ± 2.45
28	33.09 ± 6.01	40.57 ± 1.88
29	0.53 ± 0.92	25.07 ± 5.24
30	0.00 ± 0.97	0.93 ± 0.87
31	0.00 ± 0.00	9.69 ± 1.45
32	0.00 ± 0.00	0.00 ± 0.00
33	0.00 ± 0.00	0.48 ± 0.99
34	0.00 ± 0.00	1.59 ± 2.75
35	3.96 ± 3.75	24.09 ± 4.62
36	0.00 ± 0.00	3.13 ± 1.48
37	0.00 ± 0.00	0.00 ± 0.00
38	0.00 ± 0.00	4.84 ± 1.76
39	0.71 ± 1.23	3.20 ± 3.19
40	0.00 ± 0.00	20.74 ± 0.84
41	0.00 ± 0.00	0.00 ± 0.00
42	28.37 ± 1.59	40.45 ± 3.33
43	27.67 ± 7.99	41.91 ± 2.76
44	7.54 ± 3.43	22.98 ± 3.50
45	0.00 ± 0.00	1.45 ± 2.51
46	2.12 ± 2.35	2.68 ± 2.41
47	2.50 ± 0.93	1.73 ± 1.64
48	9.62 ± 3.22	31.60 ± 3.87
49	0.00 ± 0.00	1.71 ± 2.95
50	0.00 ± 0.00	0.74 ± 1.28
51	0.00 ± 0.00	0.00 ± 0.00
B1	0.85 ± 0.00	4.16 ± 1.96
B2	0.00 ± 0.00	1.94 ± 3.08
B3	1.51 ± 1.33	5.09 ± 3.06
Controls		
Saltwater	0.00 ± 0.00	0.00 ± 0.00
Potassium dichromate	100.00 ± 0.00	100.00 ± 0.00

While an assessment of a wide range of South African propolis was not previously investigated, the toxicity of propolis compounds and one South African propolis sample was previously investigated using the brine shrimp lethality assay [17]. The propolis compounds pinocembrin, galangin and chrysin, were investigated alone and in combination. The compounds were found to be non‐toxic, with toxicity lower for the combined compounds than that independently. The toxicity of the whole propolis South African sample was also non‐toxic, at 14.78% [17]. Propolis from other regions were also found to be non‐toxic. Iranian propolis was investigated for toxicity in Wistar rats and found no toxicological differences between the experimental and control groups after 48 h [32].

## 3 Conclusion

While propolis has been extensively investigated, very little attention has been paid to its antimicrobial properties against *H. pylori*, especially regarding South African propolis. This study determined the antimicrobial activity of a wide variety of South African propolis against *H. pylori* and found that the majority of ethanolic propolis extracts had good activity, comparable to that of the gold standard, Brazilian propolis. The antimicrobial results of this study show that the chemically distinct South African propolis exhibits better anti‐*H. pylori* activity than that of other global regions. The compounds pinocembrin and pinobanksin were identified in samples displaying the best anti‐*H. pylori* activity and could possibly be responsible for the activity seen. All propolis samples investigated held little to no toxicity.

This study provides novel evidence for the potential of utilizing South African propolis in apitherapy for *H. pylori* infections and related ailments and identifies compounds that could be responsible for the anti‐*H. pylori* activity that was observed. Further investigations could explore the use of propolis and the identified compounds in vivo for reducing *H. pylori* bacterial loads and gastric ulcer healing properties.

## 4 Materials and Methods

### 4.1 Propolis Sample Collection and Preparation

**FIGURE 2:**
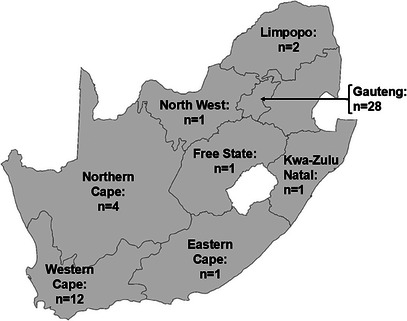
Geographic distribution of South African propolis samples used in this study (mapchart.net).

A total of 51 propolis samples (Figure 2) from various regions (dependent on beehive availability) around South Africa were donated by local beekeepers affiliated with the South African Bee Industry Organization (SABIO). Three propolis samples were obtained from Brazil (supplied by Kim Morgado) to serve as the ‘gold standard’ control. Propolis samples were stored away from light and at ambient temperature.

Before processing, samples were frozen at −20°C for 24 h. Extracts were prepared by submerging ground propolis in absolute ethanol. For antimicrobial and toxicity studies, 0.30 g/mL solutions were incubated at 37°C for 7 days with shaking at 50 rpm, followed by drying under a fume hood [7]. For chemical analysis, 1.00 mg/mL solutions were shaken for 24 h, sonicated for 10 min and filtered through a 0.22 µm syringe filter.

### 4.2 Antimicrobial Studies

Three clinically confirmed and identified isolates of *H. pylori* were obtained for this study (Dr. Ayodeji Idowu—African Institute of Digestive Diseases, Chris Hani Baragwanath Academic Hospital, Gauteng, South Africa; Ethical clearance number M210891, Human Research Ethics Committee, University of the Witwatersrand) and stored at −80°C in 20% glycerol in brain heart infusion (BHI) media.

Antimicrobial testing was carried out using the broth microdilution assay to determine the MIC, where BHI supplemented with Campylobacter Selective Supplement (Skirrow) and 7% foetal bovine serum was used. Propolis extracts were made up to 25.00 mg/mL using acetone, and a twofold serial dilution was created in 96‐well microtiter plates. Cultures of *H. pylori* made up to 0.50 McFarland turbidity standard were added to each well. The plates were sealed and incubated in a micro‐aerobic environment using an anaerobic tank and a microaerobic gas pack (CampyGen) for 24 h at 37°C. A positive control of 0.01 mg/mL amoxicillin, a negative control of acetone alone, and a culture control were included. A solution of 0.02 mg/mL *p*‐iodonitrotetrazolium violet was used to indicate the viability of cultures in each well. The MIC of each sample was taken as the lowest concentration that inhibited growth completely [33]. All assays were carried out in triplicate, and the mean and standard deviation were recorded.

### 4.3 Chemical Analysis

Samples that demonstrated the best antimicrobial activity were selected for chemical analysis using ultra‐performance liquid chromatography–quadrupole time of flight–mass spectrometry (UPLC‐QToF‐MS) (Department of Pharmaceutical Sciences, Tshwane University of Technology). Chromatographic analyses were performed on a Waters Acquity I Class Ultra‐Performance Liquid Chromatographic system coupled to a PDA detector (Waters, Milford, MA, USA). Chromatographic separation was achieved on an Acquity UPLC BEH C18 column (150 mm × 2.10 mm, i.d., 1.70 µm particle size; Waters) maintained at 40°C. The mobile phase consisted of 0.1% formic acid in water (Solvent A) and 0.1% formic acid in acetonitrile (Solvent B) at a flow rate of 0.35 mL/min. The gradient elution was as follows: 90% A: 10% B, changing to 60% A: 40% B in 2.5 min, changing to 55% A: 45% B in 8.5 min, changing to 20% A: 80% B in 1.5 min, keeping for 0.5 min, back to the initial ratio in 0.5 min and equilibrating the system for 1.5 min. The total running time was 15 min. The samples were injected in the mobile phase with an injection volume of 1.00 µL (full‐loop injection). Data were collected and processed by the chromatographic software MassLynx 4.2.

Mass spectrometry (G3 QTof, Waters) was operated in the negative ion electrospray mode. Nitrogen was used as the desolvation gas. The desolvation temperature was set to 350°C at a flow rate of 550 L/h and the source temperature was 100°C. The capillary and cone voltages were set to 2500 and 40 V, respectively. Data were collected between 100 and 1200 *m*/*z*. During acquisition, the LockSpray interface was used to ensure mass accuracy and reproducibility.

### 4.4 Toxicity Studies

The brine shrimp lethality assay was used to assess the toxicity, where *Artemia franciscana* (brine shrimp) eggs (Ocean Nutrition) were hatched in 32.00 g/L saltwater and exposed to light. Propolis extracts were made up to 1.00 mg/mL using 2% DMSO. Aliquots (400 µL) of brine shrimp in saltwater (40–60 shrimp) and propolis extracts were added in triplicate to the wells of a 48‐well micro‐titre plate. Dead shrimp were counted at 0, 24 and 48 h. Glacial acetic acid was added after 48 h, the total dead shrimp in each well were counted, and the percentage mortality was calculated. Samples with a mortality percentage greater than 50% are considered toxic. A positive control of 1.60 mg/mL potassium dichromate and a negative control of 32.00 g/L saltwater were included in each assay [34].

## Author Contributions

**Sarhana Dinat**: writing – original draft, methodology, investigation, funding acquisition, formal analysis, data curation, conceptualization. **Ané Orchard**: writing – review and editing, supervision, funding acquisition, conceptualization. **Efficient Ncube**: writing – review and editing, methodology, investigation, formal analysis. **Weiyang Chen**: writing – review and editing, methodology, investigation, formal analysis. **Alvaro Viljoen**: writing – review and editing, resources. **Sandy Van Vuuren**: writing – review and editing, supervision, software, resources, project administration, funding acquisition, conceptualization.

## Conflicts of Interest

The authors declare no conflicts of interest.

## Data Availability

The authors have nothing to report
